# Overexpression of *eIF-5A2 *in mice causes accelerated organismal aging by increasing chromosome instability

**DOI:** 10.1186/1471-2407-11-199

**Published:** 2011-05-26

**Authors:** Muhan Chen, Jian-Dong Huang, Hong Kui Deng, Suisui Dong, Wen Deng, Sze Lan Tsang, Michael SY Huen, Leilei Chen, Tong Zan, Gui-Xia Zhu, Xin-Yuan Guan

**Affiliations:** 1 Department of Clinical Oncology, Faculty of Medicine, The University of Hong Kong, 21 Sassoon Road, Hong Kong, China; 2 Department of Biochemistry, Faculty of Medicine, The University of Hong Kong, 21 Sassoon Road, Hong Kong, China; 3 Department of Cell Biology, College of Life Sciences, Peking University, Beijing, China; 4 Department of Anatomy, Faculty of Medicine, The University of Hong Kong, 21 Sassoon Road, Hong Kong

**Keywords:** *eIF-5A2*, aging, chromosome instability, transgenic mouse, oncogene

## Abstract

**Background:**

Amplification of 3q26 is one of the most frequent genetic alterations in many human malignancies. Recently, we isolated a novel oncogene *eIF-5A2 *within the 3q26 region. Functional study has demonstrated the oncogenic role of *eIF-5A2 *in the initiation and progression of human cancers. In the present study, we aim to investigate the physiological and pathological effect of *eIF-5A2 *in an *eIF-5A2 *transgenic mouse model.

**Methods:**

An *eIF-5A2 *transgenic mouse model was generated using human *eIF-5A2 *cDNA. The *eIF-5A2 *transgenic mice were characterized by histological and immunohistochemistry analyses. The aging phenotypes were further characterized by wound healing, bone X-ray imaging and calcification analysis. Mouse embryo fibroblasts (MEF) were isolated to further investigate molecular mechanism of *eIF-5A2 *in aging.

**Results:**

Instead of resulting in spontaneous tumor formation, overexpression of eIF-5A2 accelerated the aging process in adult transgenic mice. This included decreased growth rate and body weight, shortened life span, kyphosis, osteoporosis, delay of wound healing and ossification. Investigation of the correlation between cellular senescence and aging showed that cellular senescence is not required for the aging phenotypes in *eIF-5A2 *mice. Interestingly, we found that activation of *eIF-5A2 *repressed p19 level and therefore destabilized p53 in transgenic mouse embryo fibroblast (MEF) cells. This subsequently allowed for the accumulation of chromosomal instability, such as errors in cell dividing during metaphase and anaphase. Additionally, a significantly increase in number of aneuploidy cells (*p *< 0.05) resulted from an increase in the incidences of misaligned and lagging chromosomal materials, anaphase bridges, and micronuclei in the transgenic mice.

**Conclusion:**

These observations suggest that *eIF-5A2 *mouse models could accelerate organismal aging by increasing chromosome instability.

## Background

It is believed that the process of malignant tumor is a multiple-step process caused by the accumulation of abnormal expression of oncogenes and tumor suppressor genes. Therefore, the identification of commonly amplified chromosomal region and corresponding overexpressed oncogenes within the region is imperative to understand the molecular mechanism of cancer development. Amplification of chromosomal region 3q26 is frequently detected in solid tumors, including ovarian [1], lung [2], esophageal [3], prostate [4], breast [5], and nasopharyngeal cancers [6], suggesting that 3q26 contains an oncogene(s) related to the pathogenesis of human cancers. Using hybrid selection approach, we identified a candidate oncogene, eukaryotic translation initiation factor 5A2 (*eIF5-A2*), from 3q26.2 [7,8]. The functions of *eIF-5A2 *are mainly revealed in cancer initiation and progression. The tumorigenic ability of *eIF5-A2 *has been demonstrated by several *in vitro *evidences: *eIF-5A2 *stably transfected LO2 cells (immortalized human liver cell line) displayed increased colony formation in soft agar and xenograph formation in nude mice; reduction of eIF-5A2 in ovarian cancer cell line UACC-1598 inhibits cell growth; and the oncogenic ability of *eIF5-A2 *can be blocked by *eIF-5A2 *silencing [7-9]. Previously, we showed that overexpression of eIF-5A2 at the protein level was significantly associated with the advanced stages of ovarian cancer [8]. Similarly, Marchet *et al. *recently reported that overexpression of eIF-5A2 is associated with a higher risk of lymph node metastasis in human gastric adenocarcinomas [10]. Recently, Zender *et al. *identified that *eIF-5A2 *is amplified in human cancer using representational oligonucleotide microarray analysis (ROMA), and is required for proliferation of *XPO4*-deficient tumor cells and promotes hepatocellular carcinoma in mice [11]. However, the *in vivo *function of *eIF-5A2 *is still not clear.

To further investigate the functions of the *eIF-5A2 *gene *per se*, we generated *eIF-5A2 *transgenic mice. Unexpectedly, the *eIF-5A2 *transgenic mice exhibit accelerated organismal aging phenotypes instead of forming spontaneous tumors. Cancer is assumed to be a disease of accumulated aged cells because of the close link between incidence of the cancer and the aging process. For decades, age has been regarded as the largest risk factor associated with the cancer initiation, which is supported by cancer incidence rising exponentially with age [12,13]. Mechanistically, genomic instability in somatic cells has been implicated as one of the major stochastic causes of aging [14-16]. Furthermore, cellular senescence, a potential *in vitro *counterpart of organismal aging, was demonstrated by several recent studies as a barrier to tumorigenesis, and contributes to the cytotoxicity of certain anticancer agents [17]. However, it is not clear whether aging functions the same way as cellular senescence to suppress tumor initiation *in vivo*. Also, there is no direct evidence to elucidate the functional contribution of senescent cells towards the onset of aging.

In this study, we found that eIF-5A2 overexpression triggers premature aging in multiple organs of the transgenic mice. We further demonstrated that supraphysiological expression of eIF-5A2 repressed p19 expression and therefore impaired p53 levels. This allowed for the accumulation of chromosomal instability, which ultimately led to organismal aging. To our knowledge, this study revealed for the first time a role of putative oncogenic *eIF-5A2 *in accelerating the aging process. Moreover, the accelerated aging process shows tumor suppression effects *in vivo*, and cellular senescence is not required for this single genetic change caused aging.

## Methods

### Generation of *eIF-5A2 *transgenic mice

A 462 bp human *eIF-5A2 *cDNA fragment was cloned into the pCAGGS vector. The linearized constructs were injected into one-cell-stage F1 mouse embryos, which were transplanted into pseudo-pregnant females. All resulting pups were screened for the presence of the transgene using a pair of primers from the vector sequence and a pair of primers from the human *eIF-5A2 *gene. For studies relying on timed pregnancies, mating pairs were established and mice were monitored daily for vaginal plugs, the presence of which would indicate 0.5 days post-copulation. Animal experimentation was done in accordance with the guidelines of the University of Hong Kong regarding the care and use of laboratory animals.

### Western and Northern blot analysis

For Western blot analysis, Protein lysates were prepared with RIPA buffer (1 × PBS, 1% Nonidet P40, 0.5% sodium deoxycholate, 0.1% SDS and protease inhibitor cocktail). About 10 μg of lysate was separated by SDS-polyacrylamide gel electrophoresis, transferred to a PVDF Hybond-P membrane (Amersham Pharmacia Biotechnology, Piscataway, NJ), and detected by antibodies for eIF-5A2 (a mouse monoclonal antibody raised against the 54 residues of eIF-5A2), p53 (Zymed, San Francisco, CA), p21 (Upstate, Temecula, CA), p19 (Upstate, Lake Placid, NY), CDK4 (Cell Signaling Technology, Beverley, MA), and γ-tubulin (Sigma, St. Louis, MO). For Northern blot analysis, total cellular RNA was prepared using the TRIzol/chloroform method. Twenty microgram of RNA was size fractionated, transferred to a nylon membrane, and hybridized with a ^32^P-labeled human *eIF-5A2 *specific probe as described previously[7,8].

### Histological analysis, immunohistochemistry, senescence and BrdU incorporation assay

Fresh mouse tissues were fixed in 4% cold paraformaldehyde in PBS, processed into serial paraffin sections, and stained with Mayer's hematoxylin-eosin staining. Immunohistochemistry (IHC) was performed using the following antibodies: eIF-5A2 (1:500 dilution), PCNA (1:500 dilution, Santa Cruz Biotechnology, Santa Cruz, CA). Internal standardization was achieved by comparing only images stained with the same antibodies in the same experiment, captured with identical parameters, and scaled and displayed identically. For β-galactosidase staining, MEF cells were stained for senescence-associated acidic β-galactosidase activity according to the manufacturer's protocol (Cell Signaling Technology, Beverley, MA). BrdU (100 mg/g of body weight) was injected i.p. into pregnant females. Then the animals were killed 2 h after injection and the mouse tissues or embryos were fixed in 4% paraformaldehyde at 4°C overnight. Then the sections were processed for stained with BrdU staining kit (ZYMED) according to the manufacturer's protocol.

### Wound healing experiments

4-month old mice were anaesthetized with methoxyfluorane, and the dorsum was shaved and cleaned with alcohol. Three equidistant 1-cm full-thickness incisional wounds were made through the skin and panniculus carnosus muscle. Wounds were measured at days 1, 2, 3 and 4 post-wounding, and wounded skin specimens were collected and bisected for histology at day 4 post-wounding.

### Bone X-ray imaging and calcification analysis

Individual mice were subjected to an X-ray imager for the detection of kyphosis and osteoporosis. In brief, mice were anaesthetized with 2% isofluorane and images were taken by a 600P X-ray mammogram machine (General Electric Co., Albuquerque, NM) with a dose of 15 kV for 100 sec. For calcification analysis, embryos were eviscerated and the skin was removed. The embryos were fixed in 95% ethanol and stained in Alcian blue solution and Alizarin red solution overnight as described previously[18].

### Isolation of mouse embryo fibroblast (MEF) cells

E13.5 embryos were digested at 37°C for 10 min in 0.2% trypsin (Sigma, St. Louis, MO) in PBS (pH 7.4). The cell suspension was cultured in Dulbecco's modified Eagle's medium (DMEM) supplemented with 10% FBS at 37°C. For cell proliferation analysis, 5 × 10^4 ^wild-type and *eIF-5A2 *transgenic MEF cells were plated on 6-well plates and cell numbers were counted every day for up to 6 days. For flowcytometry test, cells were fixed in 70% ethanol, stained with propidium iodide, and analyzed by flow cytometer.

### Cytogenetic analysis, FISH and SKY

Metaphase spreads were prepared from either cultured MEF cells or bone marrow lymphocytes. Metaphases were harvested from cultured MEFs by Colcemid treatment (0.03 μg/ml) for 3 hr or from bone marrow lymphocytes in tested mice by colchincine treatment (3 mg/kg, intraperitoneal injection). Metaphase spreads were stained by standard trypsin-Giemsa banding method and analyzed under microscope as described previously [7].

For fluorescence *in situ *hybridization (FISH) analysis, the whole transgene construct was used as a probe, which was labeled with Spectrum Red-dUTP by nick translation (Life Technologies, Inc.). The labeled probe was then hybridized to prebanded wild-type or *eIF-5A2 *transgenic MEF metaphase chromosomes as described previously [7].

Spectral karyotyping (SKY) was performed using a SKY probe (Applied Spectral Imaging, Migdal Ha'Emek, Israel) as described previously [19,20]. The signal detection followed the recommendations of the SKY probe manufacturer. SKY image capturing and karyotyping were performed using the SkyVision Imaging System equipped with a Zeiss Axioplan 2 fluorescence microscope.

### Telomerase activity assay

The telomere repeat amplification protocol (TRAP) assay was performed using the TRAPEZE kit (Invitrogen, New York, NY) following the manufacturer's protocol. Except for negative control (lysis buffer), 0.05 μg protein was used for each PCR, which was run for 33 cycles.

### Statistical analysis

Statistical analysis was performed using the SPSS software (SPSS Standard version 8.0). Significance of difference was analyzed using Student's t tests. A significant difference was considered when the *p *value was less than 0.05.

## Results

### Generation of *eIF-5A2 *transgenic mouse lines

**Figure 1:**
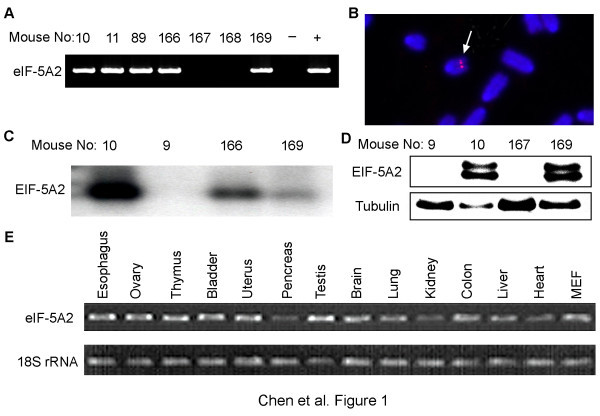
**Generation of *eIF-5A2 *transgenic mice**. (*A*) Three *eIF-5A2 *transgenic mouse founders (mouse No.: 10, 11, and 89) and their offspring (166-169) were determined by PCR. One mouse without eIF-5A2 transgene (No. 9) was used as negative control. Mouse fibroblast cell line NIH 3T3 and *eIF-5A2 *transgenic construct was used as negative (-) and positive (+) controls. (*B*) The transgene *eIF-5A2 *was mapped to one mouse chromosome site in line 11 by FISH. The metaphase spread was prepared from bone marrow lymphocyte. Arrow indicates the hybridization signals of *eIF-5A2*. (*C*) Expression of *eIF-5A2 *in transgenic mice was confirmed by Northern blot analysis. A human *eIF-5A2 *cDNA probe was used and it did not detect the endogenous *eIF-5A2 *mRNA in both wild-type and transgenic mice. (*D*) Western blot showed the overexpression of human eIF-5A2 in transgenic mice using liver lysates. The lower bands (17dD) were eIF-5A2 and the upper bands were shifted eIF-5A2 bands caused by posttranslational hypusination. (*E*) Expression of transgene *eIF-5A2 *in various tissues of transgenic mouse was detected by RT-PCR using a pair of human-specific primers.

To characterize the role of *eIF-5A2 in vivo*, we generated transgenic mouse lines overexpressing human *eIF-5A2 *ubiquitously. Pronuclear injection of the pCAGGS-*eIF-5A2 *construct resulted in the generation of three founder mice, as identified by genomic Southern blot analysis and PCR (Figure 1A). To confirm the single integration event, an *eIF-5A2 *probe was used to hybridize to the metaphase spreads from bone marrow lymphocytes by fluorescence *in situ *hybridization (FISH). The transgene was mapped to one chromosome site in line 11 (Figure 1B). All the founders were able to transmit the transgene to their offspring in the expected Mendelian ratio. Three lines were expanded, and the expression of the transgene was confirmed in two out of three founder lines (10 and 11). Expression of *eIF-5A2 *at the RNA level was detected by RT-PCR and Northern blot hybridization using liver and testis tissues (Figure 1C). Also, EIF-5A2 protein expression was demonstrated by immunohistochemistry (IHC) and Western blot analysis (Figure 1D) in multiple organs. Of note, no difference was observed in the expression of murine endogenous eIF-5A2 among the founders and wild-type controls. To ensure that the observed phenotypes were not a result of genomic insertion positional effects, we performed all experiments with both transgenic lines and obtained similar results for both lines. Expression of transgene eIF-5A2 in trnasgenic mice could be detected in all tested tissues by RT-PCR (Figure 1E).

### Characterization of *eIF5-A2 *transgenic mice

**Figure 2:**
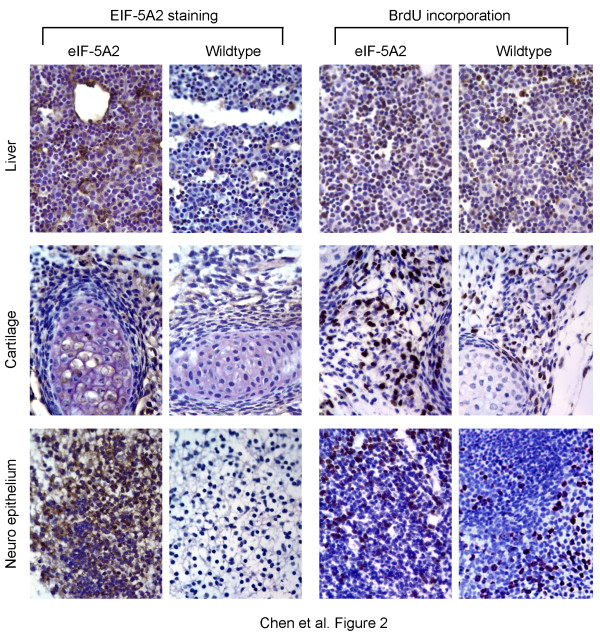
**Tissue distribution of transgenic eIF-5A2 protein and BrdU incorporation analysis of day 13.5 embryos**. Expression of eIF-5A2 in developing liver, cartilage and neuroepithilium from transgenic and wild-type embryos (left) was detected by IHC (magnification, 400 ×). BrdU incorporation assay (right) was used to compare the proliferation rates between transgenic and wild-type embryos. No significant difference was observed in the percentage of BrdU positive cells between transgenic (56.3 ± 7.81) and wild-type embryos (52.73 ± 9.14, *p *> 0.05).

**Figure 3:**
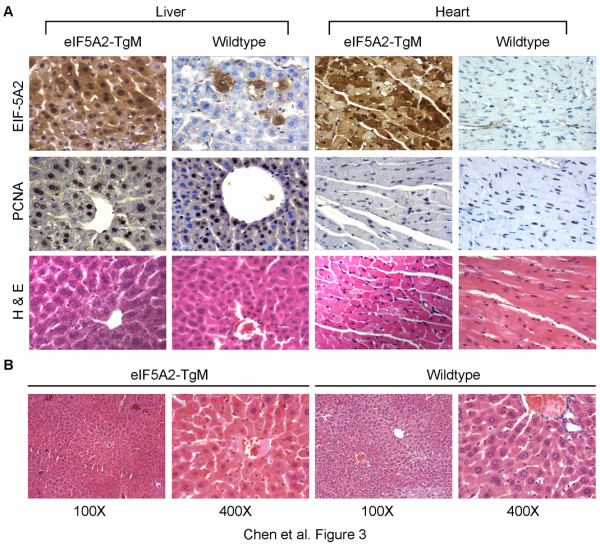
**eIF-5A2 expression, cell proliferation and histopathological analysis of 12-week-old mice **(magnification, 400 ×). (*A*) Top panel, eIF-5A2 immunostaining of liver and heart from transgenic and wild-type mice. Middle panel, proliferation was measured by using PCNA immunostaining and the percentage of PCNA positive cells in transgenic mice (46.2 ± 10.98) was comparable to that in wild-type littermates (37.87 ± 10.44, *p *> 0.05). Bottom panel, haematoxylin and eosin (H&E) staining of adult liver and heart tissues. (*B*) H&E staining of the transgenic and wild-type livers after 12-week alcohol treatment.

To determine the effects of *eIF-5A2 *on mouse development, protein expression in13.5-day embryos and 12-week old mice was studied. On embryonic day 13.5, expression of eIF-5A2 was detected in the developing brain, liver and cartilage (Figure 2). At 12-week of age, EIF-5A2 protein expression was detected in all the tested adult tissues, including brain, lung, heart, liver, kidney, spleen, testis, uterus and ovary (sure 3A). Histological analysis showed that the morphologies of all tested tissues in *eIF-5A2 *transgenic embryos/mice were indistinguishable from their wild-type counterparts. To test the effects of eIF-5A2 overexpression on cell proliferation, BrdU incorporation assay and proliferating cell nuclear antigen (PCNA) immunostaining was applied to measure the proportion of cells in S phase in E13.5 embryos and12-week-old mice, respectively. However, the frequencies of BrdU positive cells in transgenic and wild-type embryos (56.3 ± 7.81 vs. 52.73 ± 9.14, respectively; *p *> 0.05) and PCNA positive cell percentages in transgenic and wild-type mice (46.2 ± 10.98 vs. 37.87 ± 10.44, respectively; *p *> 0.05) were comparable (Figure 2 and 3A).

No spontaneous tumor formation was observed in *eIF-5A2 *transgenic mice (n = 452) during a period of 3 years. Alcohol intoxication, an efficient way to induce liver tumorigenesis in cancer-prone rodents [21], was used to induce liver lesions in 12-week-old *eIF-5A2 *transgenic mice (n = 20) and wild-type littermates (n = 12). After 13 weeks of alcohol treatment, livers of transgenic and wild-type mice were harvested and examined by histologists. No visible liver tumors or precancerous lesions were detected in all of the tested animals (Figure 3B). This suggests that eIF-5A2 overexpression alone does not increase the susceptibility of cancer onset in mice.

### Exhibition of accelerated aging phenotypes in *eIF-5A2 *transgenic mice

**Figure 4:**
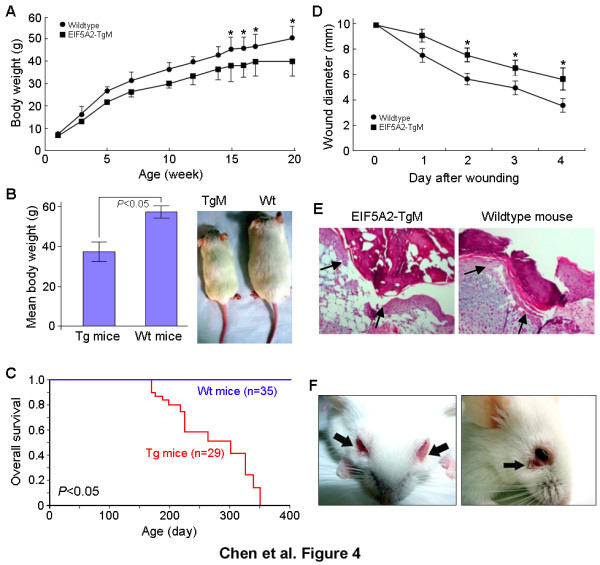
**Aging-related phenotypes in *eIF5A2 *transgenic mice**. (*A*) Cumulative plot of body weight versus age of male *eIF-5A2 *transgenic mice (n = 11) and their wild-type siblings (n = 8). * *P *< 0.05. (*B*) Quantification of the mean body weight of 5-month-old male *eIF-5A2 *transgenic mice and wild-type littermates is shown in the left (*p *< 0.05), and the representative image of a 24-week-old transgenic mouse and wild-type sibling was shown in the right. The body size of the transgenic mouse was significantly smaller than its wild-type sibling (*P *< 0.05). (*C*) Kaplan-Meier survival curve of male transgenic mice (n = 29, red line) and wild-type controls (n = 35, blue line). (*D*) Comparison of wound healing rates between 4-month-old *eIF5A2 *transgenic mice (15 wounds in 5 transgenic mice) and their wild-type siblings (12 wounds in 4 mice). * *P *< 0.05. (*E*) Representative HE-stained section at day 4 post-wounding in an *eIF-5A2 *transgenic mouse (left) and its wild-type sibling (right). The ability of re-epithelialization in the edges of wound was remarkably reduced in the transgenic mouse compared with its wild-type control (indicated by arrows). (*F*) Chronic skin lesions in *eIF-5A2 *transgenic mice (indicated by arrows).

*eIF-5A2 *transgenic mice were indistinguishable from their wild-type siblings at birth. However, phenotypes of transgenic mice and wild-type littermates could be distinguished from postnatal week 3 according to body size and weight. From 3 weeks of age, the growth rate of transgenic mice was significantly reduced (about 20-40%, *P *< 0.05, Student's *t *tests) compared with wild-type controls (Figure 4A). At 5 months of age, the body weights of transgenic male mice (n = 11) and wild-type male littermates (n = 8) were compared, and we found the mean body weight of wild-type mice (57 ± 3.08 g) was significantly higher than that of transgenic littermates (37.1 ± 4.83 g, *p *< 0.05, Student's *t *tests) (Figure 4B). Strikingly, most of the transgenic mice died at 7-9 months without any apparent causes of death or visible changes observed by autopsy. The average lifespan of eIF-5A2 mice is 8 months (Figure 4C).

### Skin aging phenotypes in *eIF-5A2 *mice

Reduction of the capacity to respond to stresses such as wound healing is often associated with human aging [22]. Wound healing ability was compared between *eIF-5A2 *transgenic mice and their wild-type siblings. We found that wound healing was significantly delayed (*p *< 0.05, Student's *t *tests) in transgenic mice compared with their wild-type littermates (Figure 4D). Histological analysis showed that the ability of re-epithelialization in the edges of the wound was remarkably reduced in transgenic mice compared to their wild-type controls (Figure 4E). In addition, chronic skin lesions frequently occurred in *eIF-5A2 *transgenic mice due to the unhealing wounds or scratches (Figure 4F).

### Skeletal degeneration in *eIF-5A2 *transgenic mice

**Figure 5:**
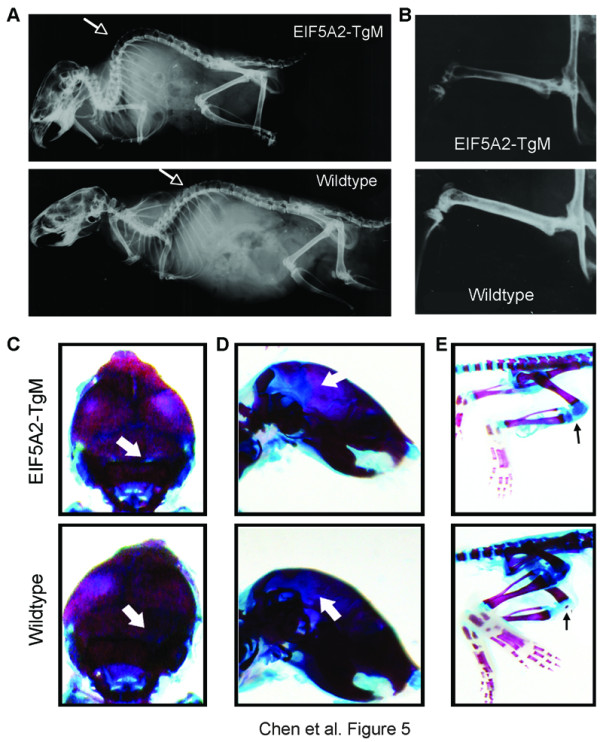
**Skeletal aging phenotypes in *eIF-5A2 *transgenic mice**. (A) X-ray radiograph was used to examine skeletal changes in 24-week-old *eIF-5A2 *transgenic mouse (upper) and their wild-type sibling (lower). Kyphosis was observed in *eIF5A2 *mouse (indicated by an arrow). (*B*) Representative radiograph of the density of femur bone in an *eIF-5A2 *transgenic mouse (24-week-old) and a wild-type mouse. (*C, D*) Alcian blue and Alizarin red staining of skulls from a 2-week-old transgenic mouse (upper) and its wild-type sibling (lower). A wider cranial sutures (*C*) and fontanelle (*D*) were observed in transgenic pup (indicated by arrows). (*E*) Representative skeletal staining of hind limb in a 2-week-old transgenic mouse (upper) and its wild-type sibling (lower). A clear ossification of patella was observed in the knee joint of wild-type mouse but not in the transgenic mouse (indicated by arrows).

Radiographs were taken to examine for potential skeletal degeneration in *eIF-5A2 *transgenic mice (n = 6) and their wild-type siblings (n = 5) at 24 weeks of age. Almost all the *eIF-5A2 *transgenic mice showed varying severity of kyphosis at 24-week of age (Figure 5A). In addition, the transgenic mice exhibited severe osteoporosis by radiographic analysis compared with their wild-type siblings (Figure 5B). We also stained the whole mouse skeletons with Alcian blue and Alizarin red to look at bone and cartilage. Interestingly, we found that 2-week-old transgenic mice showed several signs of delay of ossification. Compared to their wild-type siblings, transgenic pups had wide fontanelle and cranial sutures (Figure 5C and 5D). In the hind limb, clear ossification of the patella at the knee joint was observed in wild-type mice, but not in transgenic mice (Figure 5E). These data suggest that eIF-5A2 overexpression impairs ossification during development and contributes to osteoporosis and spine degeneration in adulthood.

### *eIF-5A2 *activation is insufficient for triggering cellular senescence

**Figure 6:**
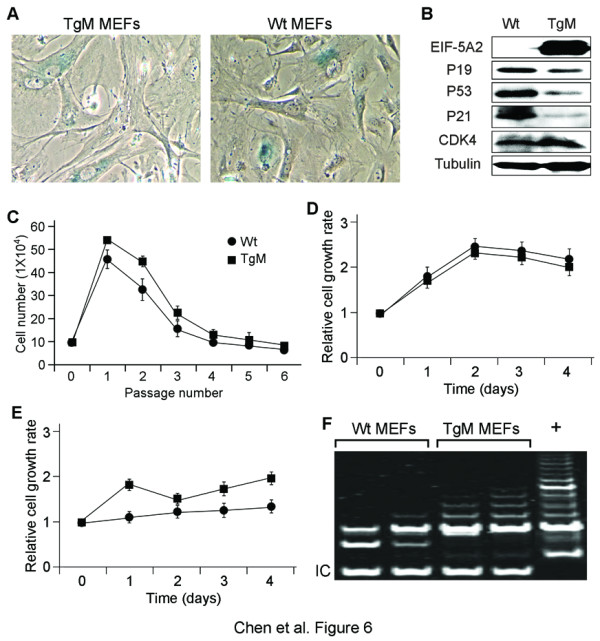
**Characterization of the *eIF-5A2 *transgenic MEF cells**. (*A*) Senescence associated β-galactosidase staining in transgenic and wild-type MEFs. No difference was observed between these two MEFs. (*B*) Western blot analysis of lysates from transgenic and wild-type MEFs showed activation of *eIF-5A2 *repressed p19 level and therefore destabilized p53 and consequently repressed p21 in transgenic MEFs. (*C*) The replication capacity of both MEFs were measured by seeding 1 × 10^4 ^cells into one well of 6-well plate and counted every 3 days. Both wild-type and transgenic MEFs proliferated normally until passage 4 and proliferation capability declined during later passages (p5 and p6). (*D*, *E*) The comparison of cell growth curves of transgenic and wild-type MEFs in 10% serum (*D*) and 1% serum (*E*). The cell growth rate was significantly higher in transgenic MEFs than in wild-type MEFs (*p *< 0.05). (*F*) Increased telomerase activity was detected in *eIF-5A2 *transgenic mice compared with their wild-type siblings. Protein extracted from HeLa cells was included as positive (+). A 36-bp internal standard was used as a control (IC) in the assay: the bands were weaker in samples with excessively high telomerase activity because amplification of the TRAP products and the IC bands were semicompetitive.

Cellular senescence can be triggered by oncogene activation and agents that damage DNA or alter chromatin structure [13,17]. Given our observation of organismal aging in *eIF-5A2 *mice, we further investigated whether cellular senescence exists under these circumstances. Therefore, MEFs from *eIF-5A2 *transgenic mice and their wild-type littermates were characterized. *eIF-5A2 *MEFs were morphologically indistinguishable from the wild-type counterparts at both early and late passages. The activity of senescence associated β-galactosidase (SA-β-gal), a cellular index of senescence, was eximined and no difference was found between *eIF-5A2 *transgenic and wild-type MEFs (Figure 6A). We also stained the cryostat sections of skin, testis and liver, and did not detect any SA-β-gal positive cells *in vivo *(not shown).

The p53 tumor suppressor pathway in senescence has been well studied and serves as a critical player that mediates both replicative and oncogene-induced senescence. Senescence plays an important role in protecting the tumorous transformation of a living cell by oncogene activation via up-regulation of p53, p21 and p19 [23,24]. We examined p53 levels and found a striking reduction of p53 at steady state (Figure 6B). Consequently, p53 transcriptionally down-regulated its downstream target p21^Cip1 ^and indeed we found less p21^Cip1 ^in *eIF-5A2 *MEFs. p19^Arf ^interferes with p53 negative regulator Mdm2 and therefore induces and stabilizes p53 [25,26]. It has been reported that the absent of p19 can enhance Mdm2's ability to down-regulate p53 during stress. We found a reduction of p19 at the protein level in *eIF-5A2 *MEFs, suggesting that *eIF-5A2 *activation serves as oncogenic stressor and thus repressing p19 (Figure 6B). Subsequently, CDK4, the down stream cell cycle molecule of p19 inhibitor, was up-regulated in transgenic MEFs (Figure 6B), suggesting that *in vitro *overexperssion of *eIF-5A2 *contributes to oncogenic transformation, but does not drive the onset of cellular senescence.

### Activation of eIF-5A2 caused partial transformation in MEFs

Due to the massive overexpression of eIF-5A2 and reduced levels of p53 in transgenic MEFs, we wondered whether these together would favor cell proliferation. Therefore, firstly transgenic and wild-type MEFs were continuously passaged and monitored the replicative capability. Both MEFs proliferated normally until passage 4 and the proliferation declined during later passages (passage 5 and 6) (Figure 6C). Next we used an XTT assay to compare cell growth rates between *eIF-5A2 *transgenic MEFs and wild-type MEFs. The cell proliferating capability of transgenic MEFs was similar to that of wild-type MEFs when fed with 10% serum (Figure 6D). However, *eIF-5A2 *transgenic MEFs grew significantly faster than wild-type MEFs in low-serum (1%) conditions (*p *< 0.05, Figure 6E).

Telomerase has been detected in human cancer cells and is more active in transformed cells than in normal cells. This provides a selective growth advantage in transformed cells. Given the facts that the activation of eIF-5A2 and down-regulation of tumor suppressors p19 and p53, we hypothesized that *eIF-5A2 *MEFs could have increased telomerase activity. We used a telomere repeat amplification assay (TRAP) to compare the telomerase activities between transgenic and wild-type MEFs. Interestingly, 6 out of 8 (75%) tested transgenic MEFs displayed increased telomerase activity compared to 7 wild-type controls (Figure 6F). Taken together, in *in vitro *culture we obtained typical, at least partial, phenotypes similar to transformed cells in transgenic MEFs overexpressing eIF-5A2, which is consistent with our previous results [7,8].

### Chromosomal instability in *eIF-5A2 *transgenic mice

**Figure 7:**
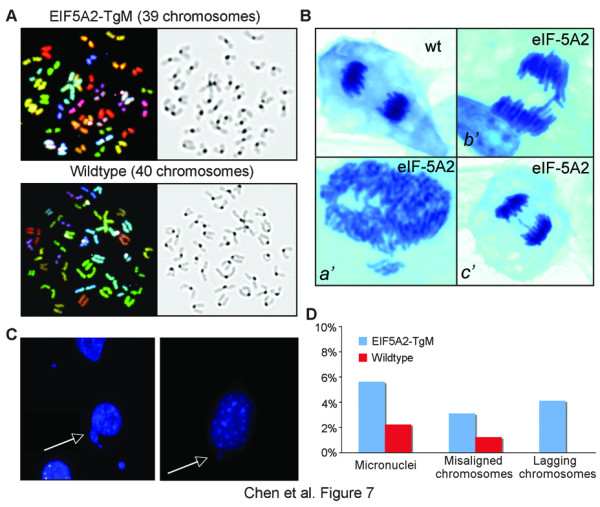
**Chromosome instability in *eIF-5A2 *transgenic mice**. (*A*) Representative SKY images (left) and reversed DAPI stain (right) of bone marrow metaphase spreads from wild-type and transgenic mice. (*B, C*) Detection of hallmarks of chromosomal instability in *eIF-5A2 *transgenic MEFs, including misaligned chromosomes separated from the metaphase plate (*a'*), lagging chromosomes in anaphase (*b' *and *c'*), and micronuclei in interphase cells (*C*). (*D*) Quantification of the incidence of the micronuclei, misaligned and lagging chromosomes.

Considering the role of p53 in maintenance of genetic stability and its down-regulation in *eIF-5A2 *transgenic mice, the correlation of eIF-5A2 expression and chromosomal instability was investigated. Metaphases from MEFs and adult mouse bone marrow were cytogenetically characterized and compared between *eIF-5A2 *transgenic mice and their wild-type littermates. Karyotyping analysis showed that the frequency of aneuploidy cells was significantly higher in transgenic MEFs (18.5%, n = 200) than that in wild-type MEFs (4.5%, n = 200, *p *< 0.05). In adult mouse bone marrow cells, the frequency of aneuploidy cells was significantly higher in transgenic mice (11.5%, n = 200) than that in wild-type mice (3%, n = 200, *p *< 0.05). Spectral karyotyping (SKY) analysis also found an increased number of aneuploidy cells in adult transgenic mice (16/100 metaphases) compared to wild-type controls (6/100 metaphases) (Figure 7A).

The dividing MEF nuclei from both *eIF-5A2 *transgenic mice and their wild-type littermates were also compared. We found a significant increase in misaligned chromosomal material in transgenic MEFs (3.1%, n = 1,000) compared to wild-type controls (1.2%, n = 1,000, *p*<0.05) (Figure 7B). Lagging chromosomal material, in the form of an anaphase bridge (Figure 7B), was detected in 4.1% (n = 1,000) of transgenic MEFs, but not in wild-type MEFs. In addition, the detection of micronuclei after mitosis was significantly higher in *eIF-5A2 *transgenic mice (5.6%, n = 1,000) than in the wild-type controls (2.2%, n = 1,000; *p *< 0.001, Figure 7C and 7D).

## Discussion

Our studies have identified *eIF-5A2 *as a single genetic change involved in accelerating the aging process in mice. Overexpression of *eIF-5A2 *results in aging phenotypes in multiple organs *in vivo*, but is insufficient for triggering cellular senescence. We provide here the mammalian genetic evidence for the notion that organismal aging is not always linked to cellular senescence.

A key finding in our study is that activation of oncogene *eIF-5A2 *represses p19 levels and impairs its stabilization of p53. Consequently, p53 transcriptionally down-regulates p21^Cip1 ^and therefore increases CDK4 in response to p19 inhibition. However, these eIF-5A2 mediated pathway alterations function very differentially in *in vivo *and *in vitro *contexts. *In vivo*, down-regulated p19, p53 and p21 and up-regulated CDK4 promote cell proliferation and increase telomere activity, which belong to oncogenic transformation category. Consequently, we observed partial transformation of *eIF-5A2 *MEFs, but not oncogene activation induced cellular senescence. In the mouse, the activation of eIF-5A2 and subsequent alteration of the p19-p53-p21 pathway, leads to accelerated aging phenotypes. This confirms again the fate of oncogene activation is context dependent. The activation of eIF-5A2 alone in an *in vitro *system shows a mild transformation capability and is insufficient to trigger the senescence response. While in the intact mice context, the stress of eIF-5A2 activation causes whole body responses and navigates to accelerated aging to suppress tumorigenesis *in vivo*. As a result, there is no spontaneous tumor formation in *eIF-5A2 *mice, and they are not cancer-prone, and they do not respond to cancer reagents.

Notably, the p19 mediated impairment of p53, allowing the accumulation of numerical genomic instability, was revealed in both MEFs and mice. Human and mouse models of accelerated aging frequently involve alterations in genome maintenance mechanisms [21]. p53 plays a critical role in cell cycle regulation and the maintenance of genetic stability, which is associated with its function in DNA damage reparation. Genomic instability has been implicated as a major causal factor in early onset of the aging phenotype, which was observed in mtr-/- mice and Ku80 null mice [27,28]. Therefore, we hypothesized that the molecular mechanism of *eIF-5A2 *in aging is associated with p53-dependent chromosomal instability. The data we collected from both *in vivo *and *in vitro *studies showed a variety of chromosomal instability, including higher incidences of unaligned and/or misaligned chromosomal materials, anaphase bridges, and micronuclei. They contribute to differential fates of either aging or transformation depending on the different contexts, confirming the curial role of genomic instability in both tumorigenesis and aging.

*eIF-5A2 *was originally identified as a proto-oncogene whose overexpression leads to cancerous transformation of hepatocellular carcinoma cell lines, and contributes to cancer progression and metastasis. However, no spontaneous tumors were detected in transgenic mice overexpressing eIF-5A2. One possible explanation could be the life span of the *eIF-5A2 *transgenic mouse is too short to allow for the accumulation of extra genetic changes, and defeat aging phenotypes, to form a detectable tumor. In conclusion, we found that activation of eIF-5A2 causes p53-mediated genomic instability and accelerates the aging phenotype in multiple tissues in mice. This finding provides more insight into the mechanism of accelerated aging caused by oncogene activation *in vivo*.

Our study also revealed that cellular senescence is not required for the organismal aging process. Cellular senescence was considered as a potential counterpart of organismal aging under certain circumstances [29-31]. Senescent cells have typical physiological changes, including large and flat morphology, higher acidic β-galactosidase enzymatic activity and profound growth defects. The growth arrest of cellular senescence is actually a tumor suppressor mechanism to resist tumorigenesis via shortening the longevity of cells and preventing cell proliferation. In addition, senescences often down-regulate extracellular matrix production and upregulate inflammatory cytokines to modulate the microenviroment or immune response [13]. Although we observed striking aging phenotypes in *eIF-5A2 *mice, there is no evidence that the aging effect of *eIF-5A2 *is associated with cellular senescence. Our model demonstrates that cellular senescence is not required for the initiation of *eIF-5A2 *mediated organismal aging. In another words, organismal aging is not necessarily the consequence of the accumulated senescent cells. This type of separation of cellular senescence and organism aging can be cell type and context dependent.

## Conclusions

In the past several decades, age has been regarded as the largest risk factor tightly linked to the initiation of cancer. Cellular senescence, a potential *in vitro *counterpart of organismal aging, contributes to the cytotoxicity of certain anticancer agents. Here, we reveal for the first time a role of putative oncogenic *eIF-5A2 *in accelerating the aging process by increasing chromosome instability, and cellular senescence is not required for the aging phenotypes in *eIF-5A2 *mice. Thus, we conclude that organismal aging is not necessarily the consequence of the accumulated senescent cells. This type of separation of cellular senescence and organism aging can be cell type and context dependent.

## Competing interests

The authors declare that they have no competing interests.

## Authors' contributions

MH, JD and HK performed the majority of experiments. WD, LL and SY carried out the cytogenetic studies. SS participated in the generation of transgenic mice. SL, TZ and GX helped with bone X-ray imaging analysis. XY supervised this project and provided suggestions. MH, SS and XY drafted and revised the manuscript. All authors reviewed, critiqued and offered comments to the text and approved the final version of manuscript.

## Pre-publication history

The pre-publication history for this paper can be accessed here:

http://www.biomedcentral.com/1471-2407/11/199/prepub
